# Long-Lasting Therapeutic Response following Treatment with Pembrolizumab in Patients with Non-Small Cell Lung Cancer: A Real-World Experience

**DOI:** 10.3390/ijms24065938

**Published:** 2023-03-21

**Authors:** Walid Shalata, Jeremy Zolnoorian, Gabrielle Migliozzi, Ashraf Abu Jama, Yulia Dudnik, Ahron Yehonatan Cohen, Amichay Meirovitz, Alexander Yakobson

**Affiliations:** 1 The Legacy Heritage Center & Dr. Larry Norton Institute, Soroka Medical Center, Ben Gurion University, Beer Sheva 84105, Israel; 2 Medical School for International Health, Ben Gurion University of the Negev, Beer Sheva 84105, Israel

**Keywords:** pembrolizumab, Keytruda, lung cancer, non-small cell lung cancer, PD-1, immune checkpoint inhibitors

## Abstract

Immune checkpoint inhibitors (ICIs), pembrolizumab in particular, have been shown to be vastly more efficacious than traditional cytotoxic or platinum-based chemotherapies in the treatment of non-small cell lung cancer (NSCLC). While there are plenty of data showing their efficacy and safety profiles, very little exists about the long-term effects of pembrolizumab. We compiled all patients with NSCLC who were treated with pembrolizumab at our institution and had progression-free survival (PFS) of at least 2 years during or after the treatment period. Within this group, we examined the long-term rates of PFS and overall survival (OS), side effect profiles, treatment, and overall disease course up to 60 months after starting treatment. This study included 36 patients with median (range) follow up times from treatment initiation in months as follows: 36 (28–65) overall; 39.5 (28–65) for adenocarcinoma; and 36 (30–58) for squamous cell carcinoma. The median (range) of OS and PFS (months) was comparable for adenocarcinoma, 36 (23–55); and squamous cell carcinoma, 35.5 (28–65). Overall, pembrolizumab shows remarkable long-term safety and efficacy in NSCLC patients. In patients who show an initially strong response and can make it to 24 months of PFS, disease progression after this period seems increasingly unlikely.

## 1. Introduction

Lung cancer is one of the most common cancers and remains the leading cause of cancer-related deaths in the USA and worldwide, with an estimated 1.8 million new cases each year [1,2,3]. The 5-year survival rate of lung cancer in the United States in 2019 was 19.4%, with the prognosis heavily influenced by the stage at which diagnosis is made [4]. The majority of cases are diagnosed once cancer has metastasized beyond the lung [5]. Over 70% of lung cancer patients diagnosed are over 70 years old, and around 3% are diagnosed under the age of 45 years [1,3]. Non-small cell lung cancer (NSCLC) is the predominant form of lung cancer, accounting for about 80% of all lung cancer cases. Histologically, NSCLC is divided into adenocarcinoma, squamous cell carcinoma (SCC), large cell carcinoma, and adenosquamous [1,5,6]. The high morbidity and mortality associated with NSCLC are caused by diagnosis at an advanced stage (approximately 70% of patients), which results in a poor prognosis at the late stage that the disease is usually caught [6,7]. The treatment of NSCLC depends on the stage of cancer and the overall health of the patient. It is important to recognize that staging holds significance as to whether the tumor can be resected. Stages I and II are localized diseases that can be resected without fear that the tumor has already metastasized, whereas in stages IIIB and IV, resection is unfeasible. Stage IIIA is unique in that T3N0M0 is resectable, whereas T3N2M0 is unresectable [6,7]. For advanced-stage NSCLC (stage 3 is unresectable), a combination of chemotherapy and radiation therapy is often the recommended treatment [8]. Traditionally, systemic chemotherapy with platinum-based formulations was the only option [7,8,9]. Outcomes for the advanced-stage disease have been poor, with a median survival period of 1 year using platinum-based treatment [7]. In recent years, immune checkpoint inhibitors (ICIs) have been used in the treatment of adjuvant, locally advanced, and metastatic diseases.

Current immune checkpoint inhibitor (ICI) therapy works by blocking the interaction between the programmed death-ligand 1 (PD-L1) on tumor cells and the programmed cell death protein 1 (PD-1) on T cells, thereby allowing the T cells to kill the tumor cells. Recent studies have proved that ICI monotherapy, or in combination with chemotherapy, improved survival, with manageable toxicity, in a subset of advanced NSCLC patients; it uses drugs to target specific mutations in the cancer cells and has become an important option for treating advanced NSCLC [2,5,10]. Pembrolizumab is currently one of the most promising checkpoint inhibitor immunotherapies. Pembrolizumab is a humanized IgG4 monoclonal antibody targeting PD-1 [11], which is used as monotherapy vs. chemotherapy and in combination with chemotherapy vs. chemotherapy alone for treating metastatic NSCLC [12,13,14,15].

The PD-1 receptor is an inhibitor of the cytotoxic T-cell response, where the binding of PD-1 to either of its ligands, PD-L1 or PD-L2, effectively turns off this immune pathway [16,17]. Certain lung cancers exhibit mutations that lead to an increased level of PD-L1 or PD-L2 expression, allowing for the evasion of the native immune system. Higher rates of PD-L1/L2 expression by cancer cells have been correlated to a poorer prognosis [18]. Pembrolizumab promotes tumor recognition by activating T cells. In patients with PD-L1 expression of at least 50%, pembrolizumab is associated with longer progression-free survival (PFS) and overall survival (OS) than the traditional platinum-based therapy [13]. Since its introduction, pembrolizumab has quickly been promoted from second- to first-line therapy for NSCLC with PD-L1 expression [19]. Studies on the efficacy of pembrolizumab have mainly used platinum-based and other combination chemotherapies as comparators, and while this ICI has shown significant increases in PFS and OS, there are limited data regarding long-term outcomes, particularly post-treatment completion. Moreover, real-world treatment results have scarcely been reported. Here, we report on 36 patients with NSCLC who were treated with pembrolizumab and their corresponding PFS, OS, and adverse effect outcomes, to add to this relatively limited pool of data.

## 2. Results

**Figure 1:**
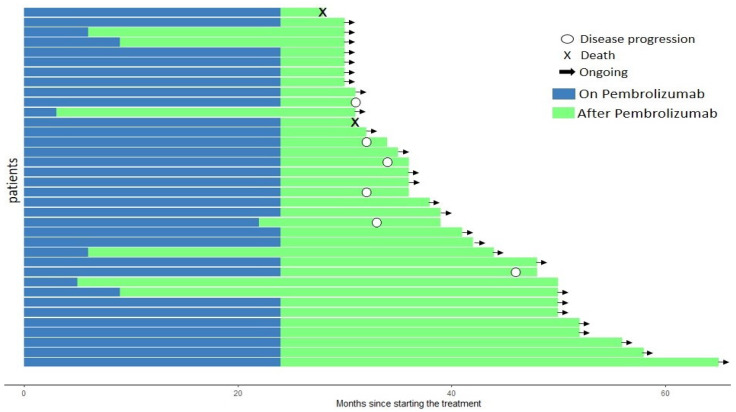
Overall survival and progression-free survival in the intention-to-treat population.

Our search yielded 36 patients who were analyzed. The median (range) of the follow-up time since starting treatment, in months, was 36 (28–65), 39.5 (28–65), and 36 (30–58) for all patients, adenocarcinoma, and squamous cell carcinoma, respectively (Figure 1). The median (range) of both OS and PFS, in months, was similar across groups: 36 (23–55) and 35.5 (28–65), respectively. The incidence of adverse events was 61.1% for all patients and similar in subgroups: 63.6% and 54.5% for adenocarcinoma and squamous cell carcinoma, respectively.

**Figure 2:**
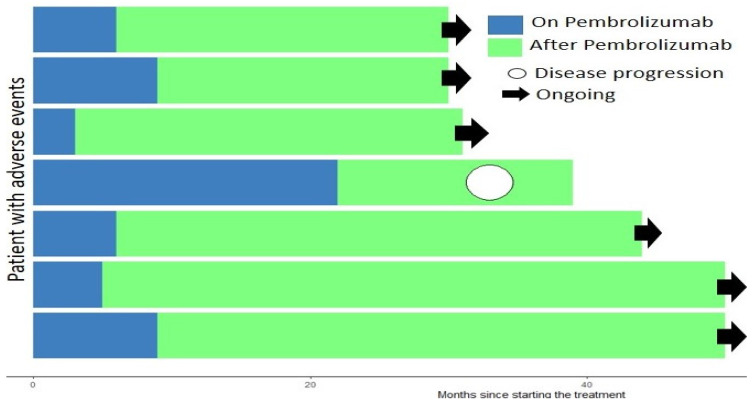
Overall survival and progression-free survival for patients who had treatment stopped early due to adverse effects.

Seven patients, 19.4% of the cohort, had to stop treatment early due to severe treatment-related adverse events. Although these patients’ total treatment time ranged from 4 to 22 months (median 14), they were kept in the study as they all showed a prolonged response with a minimum PFS of 30 months (Figure 2).

**Figure 3:**
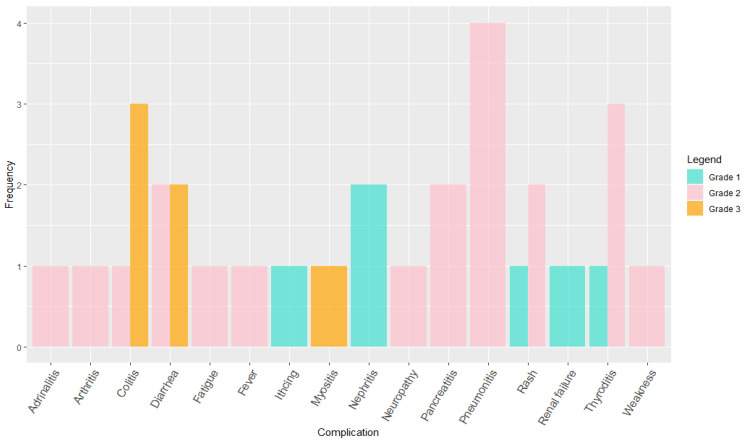
Adverse events of any cause in the as-treated population.

Only 2 of our 36 patients died during the course of our study, both due to COVID-19 infection. Six patients had disease progression (disease relapse), among whom five patients had their disease relapse in the lungs, and the sixth in the bones, while the remaining 28 patients remained progression-free at the study cut-off time (31 December 2022). It is very important to note that while all patients were treated with pembrolizumab, the majority (77.8%) were treated with additional therapy (as mentioned earlier, chemotherapy was given depending on the diagnosis: pemetrexed for adenocarcinoma and giant cell carcinoma; paclitaxel for squamous cell carcinoma and adenosquamous; patients mostly also received platinum-based treatment (69.4% of total patients)). The degree of partial vs. complete response to pembrolizumab as a first-line treatment, as well as the time taken in months to achieve a response in each patient, is visualized in Figure 3.

## 3. Discussion

The results of this study are in line with the current literature on pembrolizumab treatment of NSCLC and further support its use as a first-line agent in lung cancer with varying PD-L1 expression levels, without a treatable mutation. Pembrolizumab use continues to be associated with a relatively high efficacy profile, enhancing OS and PFS, with only a slightly higher risk of adverse events.

Previous trials in the KEYNOTE studies showed median PFS in the range of 6.9 to 10.3 months and OS in the range of 16.7 to 22.5 months [20,21,22]. Our study shows that in patients who are able to have a full 24 months without any disease progression, the longer-term outcome is remarkable. Furthermore, even another 24 months after treatment resolution, the vast majority of patients showed no progression whatsoever, with few adverse effects reported.

**Figure 4:**
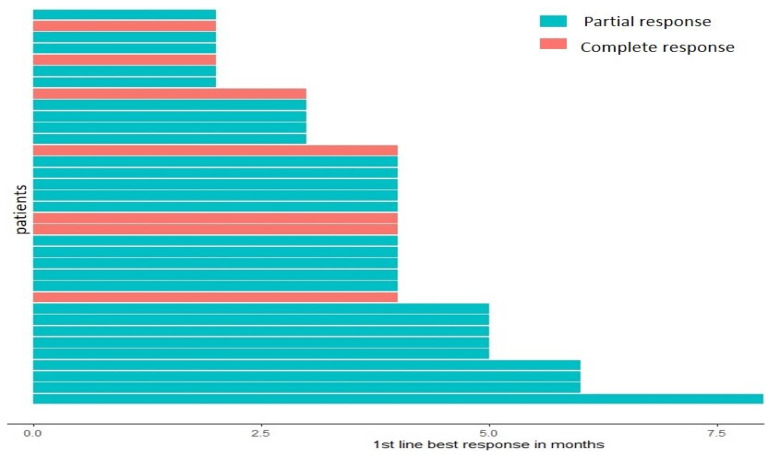
Time to best response.

The rates of adverse events were comparable to other studies on pembrolizumab: 61% in our cohort, compared to 73.4% and 63% in the KEYNOTE-24 and 42 trials, respectively [20,21]. The statistical analysis was carried out according to (1) and (2) with α = 0.05 assumed. It was determined that there was no statistically significant difference in either case. The values were $\left|Q\right|$ = 1.525 and [*Q*] = 1.96 in comparison with our data and the KEYNOTE-24 data and $\left|Q\right|$ = 0.246 and [*Q*] = 1.96 in comparison with our data and the KEYNOTE-42 data. So, the hypothesis on the absence of a significant difference was confirmed in both cases because $\left|Q\right|$ < [*Q*]. Pneumonitis (four patients, 11.1%), followed by thyroiditis (three patients, 8.3%), and colitis (three patients, 8.3%) were the most common complications of treatment. While pneumonitis was slightly more common, patients reported more complications with colitis, grade 3 vs. grade 2 for pneumonitis and thyroiditis. It is important to note that overall, the burden of severe treatment-related adverse events was relatively low; only five patients (13.9%) reported grade 3 or higher events (Figure 4). This is in comparison to 18% in the KEYNOTE-42 and 26.6% in the KEYNOTE-24 trials [20,21]. The implementation of Formulas (1) and (2) gave $\left|Q\right|$ = 0.711 for KEYNOTE-42 and $\left|Q\right|$ = 2.203 for KEYNOTE-24. [*Q*] in both cases was 1.96 with α = 0.05. So, the hypothesis that the difference was not statistically significant was confirmed for KEYNOTE-42 and rejected for KEYNOTE-24. It can be noted that the sign of *Q* was-in both cases. Therefore, there were fewer patients with a high level of complications in our cohort than in the KEYNOTE-42 and KEYNOTE-24 cohorts.

In the present study, seven patients stopped treatment early due to adverse effects. Interestingly, these patients still showed a suitable and sustained response to treatment despite suboptimal and varying treatment regimens (Figure 2). The significant and rapid therapeutic response of the tumors to immunotherapy highlights the already existing question of whether the early occurrence of immune-related adverse events may be a predictive factor of improved tumor response to immunotherapy [22,23,24]. Only one of these patients showed any disease progression during the length of study follow-up, with a PFS of 33 months, 6 months after stopping treatment. In the most extreme case, we had a single patient with squamous cell carcinoma, who only received 4 months of treatment with pembrolizumab and carboplatin, and in our last follow-up, 50 months later, was still progression-free. As far as we are aware, such prolonged responses to pembrolizumab and PFS in NSCLC patients have not been reported in the literature.

We had two patients with lung cancer histologies not included in the KEYNOTE-24, KEYNOTE-42, KEYNOTE-189, or KEYNOTE-407 trials [12,13,14,15]. There was one patient each with adenosquamous, adenocarcinoma with neuroendocrine features, and giant cell carcinoma. All three patients had PFSs of at least 30 months and at the time of the final follow up were still alive. In particular, we note that both the adenocarcinoma with neuroendocrine and giant cell carcinoma patients underwent extremely limited treatment due to adverse effects, with treatment times of 4 and 9 months, respectively. While these are only individual cases, they are noteworthy, and future studies could examine the possible treatment of these lung cancers with pembrolizumab.

The PFS for subgroups (a) with adenocarcinoma and (b) with other cancer types (including a combination of adenocarcinoma and others) was compared according to (3). As a result, the hypothesis on the absence of the statistical significance of the difference was confirmed with $\left|K\right|$ = 0.32; [*K*] = 1.75; and α = 0.05 because $\left|K\right|$ < [*K*].

The overall survival of our cohort was the most surprising aspect. The only two patients who died did so due to external complications from COVID-19. While it is entirely possible that they were at greater risk to COVID-19-induced complications and death because of their cancer, at the time of their death, there was no reason to make this assumption.

As observed during the conclusion of the final results, there were several baseline predictors of long-term outcomes in patients with advanced, metastatic lung cancer treated with pembrolizumab. The majority of the study cohort were male at 63.9%; and a good performance status (ECOG 0) was observed in 94.4% of the patients (Table 1). While a better survival rate for the pembrolizumab-combination therapy was observed across all categories of PD-L1 expression, it was noted that the higher the expression levels, the better the chances for positive long-term outcomes. A total of 69.4% of patients had PDL-1 expression >50%, 16.7% had PDL-1 expression from 1 to 49%, and 11.1% had PDL-1 expression of less than 1%, also corresponding to the lowest overall survival (Table 2).

The PFS for subgroups (a) with PDL-1 expression >75% and PDL-1 expression >50% and (b) with PDL expression <1% and 1–49% was compared according to (3). The result was that the hypothesis on the absence of the statistical significance of the difference was confirmed with $\left|K\right|$ = 0.807, [*K*] = 1.77, and α =0.05 since $\left|K\right|$ < [*K*].

Patients with a histologically confirmed adenocarcinoma (60%) showed the longest survival time. It must, however, be noted that the addition of pembrolizumab to induction therapy with pemetrexed and a platinum-based drug, as well as to pemetrexed maintenance therapy, and the addition of pembrolizumab to the standard chemotherapy with carboplatin and paclitaxel resulted in improved long-term overall survival and progression-free survival across all types of NSCLC. Other factors such as the patients’ smoking status at diagnosis and lymph node and contralateral lung involvement at diagnosis did not negatively affect the survival outcomes.

The limitations of this study were that it was performed at a single institution with a relatively small sample size. Future studies using data from multiple institutions or countries and larger patient cohorts should be performed to validate these findings. Since the inclusion criteria of this study required a minimum PFS of 24 months, patients with a lower PFS were not considered. It may be of interest to compare these two PFS groups in the future for potential differences to predict patient outcomes and improve treatment protocols; furthermore, another cohort for patients who received another type of immunotherapy may be more important. In addition, we note the lack of previous real-world studies that could be compared with our study.

## 4. Material and Methods

### 4.1. Patient Selection

This was a single-institution, retrospective observational study without intervention. Patients were selected by their electronic medical records, and participants included all NSCLC patients who were treated with pembrolizumab single-agent or combination therapy, and the selection dates ranged from August 2016 to September 2022. The last date of follow-up was 31 December 2022. The corresponding patient data were compiled into a table with the treatment regimen, start and end date of therapy, date of last follow-up, OS and PFS time, PDL status, and adverse effects. The study was approved by the Institutional Review Board of Soroka Medical Center (approval no.0316; on 8 December 2022).

### 4.2. The Inclusion Criteria for the Study

Patients aged 18 years or older.

Patients diagnosed with lung cancer: advanced or metastatic disease (stage 4).

Patients treated with pembrolizumab single-agent or combination therapy as the first-line therapy (were tested and found to be without mutation for EGFR, ALK, and ROS, as accepted in that period for the standard of care for first-line therapy).

Patients who had PFS of more than 2 years within the treatment.

All Eastern Cooperative Oncology Group (ECOG) performance-status scores of 0 to 4 (on a 4-point scale, with higher scores indicating increasing disability).

The patients had not received any previous systemic therapy for advanced or metastatic disease.

Patients must have been treated only in Soroka Medical Center or have a full follow-up history in Soroka Medical Center’s records.

Each of the studied patients was presented to and discussed with a multidisciplinary medical team when admitted to Soroka Medical Center’s Oncology Institute as per the standard protocol. This team includes a general medical and radiation oncologist, an imaging and nuclear physician, a pulmonologist, a pathologist, and a thoracic surgeon. The team discussion is based on the patient’s status, pathology, and imaging. Each patient is assigned a primary physician who is responsible for the treatment course.

Patients with previous advanced or metastatic diagnoses are treated mainly by medical oncologists, and the treatment plan is generally based on National Comprehensive Cancer Network (NCCN) recommendations [25]. Routine molecular profiling is performed for each patient when possible.

We identified 36 patients who were eligible for this study (Table 1, Table 2, Table 3 and Table 4).

### 4.3. Treatment Received

#### 4.3.1. Adenocarcinoma, Giant Cell, and Adenocarcinoma with Neuroendocrine Features

Most of the patient cohort received 4 cycles of chemotherapy of the physician’s choice. Cisplatin (75 mg per square meter of body-surface area) or carboplatin (area under the concentration–time curve, 6.5 or 4 mg per milliliter per minute (depending on performance status)), plus pemetrexed (500 mg per square meter), plus pembrolizumab (200 mg) were administered intravenously every 3 weeks. This was followed by pemetrexed (500 mg per square meter) and pembrolizumab (200 mg) every 3 weeks up to 35 cycles or unacceptable toxicity or disease progression. All patients were premedicated with folic acid, vitamin B12, and glucocorticoids according to the local guidelines for pemetrexed use. Alternatively, pembrolizumab (200 mg) was administered intravenously alone on day 1 and then every 3 weeks for up to 35 cycles or unacceptable toxicity or disease progression.

#### 4.3.2. Squamous Cell Carcinoma Adenosquamous Patients

Most of the patient cohort received 4 cycles of carboplatin (at a dose calculated to produce an area under the concentration–time curve of 6.5 or 4 mg per milliliter per minute depending on performance status) on day 1 and either paclitaxel (175 mg per square meter of body-surface area) plus pembrolizumab of 200 mg administered intravenously on day 1 and then every 3 weeks, followed by pembrolizumab (200 mg) every 3 weeks up to 35 cycles or unacceptable toxicity or disease progression; or pembrolizumab (200 mg) alone intravenously on day 1 and then every 3 weeks for up to 35 cycles or unacceptable toxicity or disease progression.

### 4.4. Data Analysis

A set of typical statistical problems were solved in the data analysis. Various small samples were compared with each other. They were either numerical (for example, PFS in months) or binary (for example, presence of adverse events, Yes/No or 1/0). In both cases, the scheme of the analysis included a check of the null-hypothesis that there was no significant difference between the parameters of the compared samples. The alternative hypothesis was one-sided, and it was that one of the sample’s average values was more than the other. A more detailed description of the methods used for biomedical problems was presented in [26].

The method of comparing binary samples was based on the properties of binomial distribution.

The final formula for the calculated criteria value of the significance of the difference (between two binary samples) is

(1) $$Q=\frac{p_{1}^{*}-p_{2}^{*}}{\sqrt{\frac{p_{1}^{*}(1-p_{1}^{*})}{n_{1}}+\frac{p_{2}^{*}(1-p_{2}^{*})}{n_{2}}}}$$

Here, $p_{1}^{*}$ and $p_{2}^{*}$ are the “frequencies” of the binary value «1» in the compared samples; *n*_1_ and *n*_2_ are the numbers of the sample elements. The modulus of the calculated value $\left|Q\right|$ (i.e., *Q* without the sign) is compared with the critical meaning [*Q*], which is detected from Formula (2):

(2) $$\left[Q\right]=\left[Q\right](\alpha )=G_{}^{-1}(\frac{1+\alpha }{2})$$

where $G_{}^{-1}$ is the inverse function for the Gauss probability distribution function and α is the assumed probability of rejection of a true hypothesis. It was taken as α = 0.05 for all calculations presented below. It corresponds to [*Q*] = 1.96. So, the hypothesis on the absence of statistically significant difference is confirmed with the probability of error α = 0.05 if $\left|Q\right|$ < [*Q*].

The quantitative samples were compared as an estimation of the statistical significance of the difference of the average values. So, the hypothesis about the equality to zero of the difference was checked. This problem does not have an exact solution when the sample volumes and the general dispersions are not equal and the general dispersions are unknown. However, there are many rough solutions based on sample dispersion estimation. The Cochran and Cox criterion was taken in this study. The calculated value of the criterion is determined in Formula (3):

(3) $$K=\frac{1}{s}\left(\bar{x}-\bar{y}\right)$$

where

(4) $$s=\sqrt{\frac{s_{1}^{2}}{n_{1}}+\frac{s_{2}^{2}}{n_{2}}}s_{1}^{2}=\frac{1}{n_{1}-1}\overset{n_{1}}{\underset{i=1}{\Sigma }}{\left(x_{i}-\bar{x}\right)}^{2}s_{2}^{2}=\frac{1}{n_{2}-1}\overset{n_{2}}{\underset{i=1}{\Sigma }}{\left(y_{i}-\bar{y}\right)}^{2}$$

Here,

$\bar{x}$ is the average value of sample 1; $\bar{y}$ is the average value of sample 2;

$n_{1}$—number of elements in sample 1; $n_{2}$—number of elements in sample 2;

$s_{1}^{2}$—dispersion in sample 1; $s_{2}^{2}$—dispersion in sample 2;

*s* —dispersion of the difference between the average values of the compared samples.

The critical value [*K*] of the criterion is determined according to the assumed α and degree of freedom number (*n* − 1) according to the t-distribution proposed by Student (W. Gosset). The null-hypothesis on statistical equivalence is confirmed if $\left|K\right|$ < [*K*].

It is important to note that confirmation of the null-hypothesis according to (1) or (3) does not mean equivalence of the compared groups. It means only that the analyzed data sets do not allow the contrary view to be maintained. Expansion of the analysis on additional data can change the conclusion.

## 5. Conclusions

This retrospective analysis of NSCLC patients who received treatment with pembrolizumab reinforces this drug’s efficacy and safety, most notably in the long term. Once patients complete a full 24-month long course of pembrolizumab, and in certain cases less than that, highly promising progression-free and overall survival statistics are seen. Furthermore, being male, PDL-1 overexpression, adenocarcinoma histological typing, and a good performance status are all positive predictors of long-term survival outcomes. Data examining pembrolizumab-treated patients several years post-treatment completion is severely lacking, and it was thus the aim of this study to contribute to this limited pool. Statistical analysis detected that in this pool, the number of severe treatment-related adverse events in the long period after pembrolizumab treatment was less than in two months in the previously conducted KEYMOTE-24 study, and the significance of the difference was confirmed statistically. A similar point relative to OS was obvious (the absence of deaths apart from two cases because of COVID-19). However, the statistical analysis displayed that the volume of used data sets is not sufficient to argue cogently that the factors enumerated above are good predictors. Additional real-world data are required for the validation of these observations and to optimize patient prognosis and predicted treatment outcomes.

## Figures and Tables

**Table 1:** Demographic and disease characteristics of patients at baseline and additional mutations present in the sample.

Age, median (range)	65.1 (46–83)
*male*	65.5 (46–85)
*female*	64.9 (53–71)
Sex	
*male*	23 (63.9%)
*Female*	13 (36.1%)
Smoking status at diagnosis	
*Current*	15 (41.6%)
*Former*	15 (41.6%)
*Never*	5 (16.8%)
ECOG status	
*0*	34 (94.4%)
*1*	2 (5.6%)
Mode of biopsy	
*Tissue*	38 (100%)

**Table 2:** Disease characteristics.

Mode of Biopsy	
*Tissue*	38 (100%)
Histology	
*Adenocarcinoma*	22 (60%)
*Squamous*	11 (31.6%)
Other *	3 (8.3%)
Lymph nodes—extrathoracic at diagnosis	26 (72.2%)
Contralateral lung affected	25 (69.4%)
Bone metastasis at diagnosis	14 (33.3%)
Adrenal metastasis at diagnosis	6 (16.6%)
Brain metastasis at diagnosis	4 (11.2%)
Liver metastasis at diagnosis	1 (2.8%)
Pleural effusion at diagnosis	1 (2.8%)
Pericardial effusion at diagnosis	1 (2.8%)
Spleen metastasis at diagnosis	0 (0%)
Peritoneal metastasis at diagnosis	0 (0%)

* Adenosquamous, Adenocarcinoma with neuroendocrine features and Giant cell carcinoma.

**Table 3:** PD-L1 expression levels at diagnosis.

*PDL1 Expression (%)*	>75%	>50%	1–49%	<1%	Not Performed
*Adenocarcinoma*	4	14	2	1	1
*Adenosquamous*	0	0	1	0	0
*Adenocarcinoma with* *neuroendocrine features*	0	0	1	0	0
*Giant cell carcinoma*	0	1	0	0	0
*Squamous cell carcinoma*	3	3	2	3	0
Total (%)	*7 (19.4%)*	*18 (50%)*	*6 (16.7%)*	*4 (11.1%)*	*1 (2.8%)*

Note: The tests were verified by immunohistochemistry from tissue biopsy. The tumor’s PD-L1 expression was measured using Ventana’s XT Benchmark using IHC PharmDx (clone 22C3, Dako), UltraView detection kit (FDA approved, Ventana).

**Table 4:** Other mutations present in the sample.

kras (G12C)	3 in adenocarcinoma
kras (G12D)	1 in adenocarcinoma
braf (V600G)	1 in adenocarcinoma
braf (G466E)	1 in adenocarcinoma
FGFR3 (Phe 384 Leu)	1 in adenocarcinoma
braf (G469V)	1 in adenosquamous
kras (G13D)	1 in squamous cell carcinoma
FGFR2 (1144T>G)	1 in squamous cell carcinoma
PTEN (209+5G>A)	1 in squamous cell carcinoma

## Data Availability

Data are contained within the article or are available from the authors upon reasonable request.
